# RNA sequencing reveals small RNAs differentially expressed between incipient Japanese threespine sticklebacks

**DOI:** 10.1186/1471-2164-14-214

**Published:** 2013-04-02

**Authors:** Jun Kitano, Kohta Yoshida, Yutaka Suzuki

**Affiliations:** 1 Ecological Genetics Laboratory, National Institute of Genetics, Yata 1111, Mishima, Shizuoka 411-8540, Japan; 2 PRESTO, Japan Science and Technology Agency, Honcho Kawaguchi, Saitama 332-0012, Japan; 3 Department of Medical Genome Sciences, the University of Tokyo, 5-1-5 Kashiwanoha Kashiwa, Chiba 277-8562, Japan

**Keywords:** Stickleback, Speciation, Variation, miRNA, piRNA, Ecology, Variation

## Abstract

**Background:**

Non-coding small RNAs, ranging from 20 to 30 nucleotides in length, mediate the regulation of gene expression and play important roles in many biological processes. One class of small RNAs, microRNAs (miRNAs), are highly conserved across taxa and mediate the regulation of the chromatin state and the post-transcriptional regulation of messenger RNA (mRNA). Another class of small RNAs is the Piwi-interacting RNAs, which play important roles in the silencing of transposons and other functional genes. Although the biological functions of the different small RNAs have been elucidated in several laboratory animals, little is known regarding naturally occurring variation in small RNA transcriptomes among closely related species.

**Results:**

We employed next-generation sequencing technology to compare the expression profiles of brain small RNAs between sympatric species of the Japanese threespine stickleback (*Gasterosteus aculeatus*). We identified several small RNAs that were differentially expressed between sympatric Pacific Ocean and Japan Sea sticklebacks. Potential targets of several small RNAs were identified as repetitive sequences. Female-biased miRNA expression from the old X chromosome was also observed, and it was attributed to the degeneration of the Y chromosome.

**Conclusions:**

Our results suggest that expression patterns of small RNA can differ between incipient species and may be a potential mechanism underlying differential mRNA expression and transposon activity.

## Background

Recent progress in the development of genomic techniques, including next generation sequencers, has greatly facilitated transcriptome analysis of ecologically important animals to reveal variations in mRNA expression patterns among closely related species and ecotypes within species [1-3]. Divergence in mRNA expression patterns is known to contribute to phenotypic evolution [4,5], although amino acid alterations in proteins are also important [6]. While a great deal is known about variation in mRNA expression profiles, information regarding naturally occurring variation in the expression patterns of small RNAs is limited, except for a few cases in plants [7,8] and cichlids [9].

Non-coding small RNAs, ranging from 20 to 30 nucleotides in length, mediate the regulation of gene expression [10-13]. The members of one class of small RNAs, microRNAs (miRNAs), are typically 20–24 nucleotides long and are highly conserved across diverse taxa [11,12]. miRNA post-transcriptionally regulates messenger RNA (mRNA). A miRNA interacts with ten to hundreds of target mRNAs to induce degradation or suppress translation [12]. Another function of miRNA is epigenetic modification of genomic DNA: miRNAs interact with target DNAs to alter the chromatin state and suppress mRNA transcription [14]. miRNAs comprise more than 1% of animal genes [15,16], suggesting that they play important roles in many biological processes. Recent functional studies in laboratory model animals such as mice, flies, and nematodes have demonstrated that miRNAs are important for regulating development, growth, pathogen resistance, and neural functions [11,12,17-19].

Another class of small RNAs is the Piwi-interacting RNAs (piRNAs), which are typically 24–32 nucleotides long and interact with Piwi proteins to suppress the expression of transposons and other functional genes [13,20]. piRNAs often possess uridine at the 5’-end (5^′^U) [13,20]. piRNAs are expressed from intergenic repetitive elements, active transposons, and piRNA clusters. Importantly, piRNAs may contribute to hybrid dysgenesis [21,22]. For example, some *Drosophila* strains contain transposons as well as piRNAs that inhibit transposon activity, whereas other strains lack both transposons and inhibitory piRNAs. Because piRNAs are maternally transmitted, hybrid progeny resulting from a cross between a mother lacking both transposons and piRNAs and a father possessing both will inherit the transposons, but not the inhibitory piRNAs. This abnormal activity of transposons in the germ line is likely to result in sterility [21,22]. Thus, maternally transmitted piRNAs can explain why hybrid abnormalities are observed in only one direction of the inter-strain crosses. piRNAs are expressed not only in the gonads, but also in the brain, and they may be involved in the regulation of neuronal functions [23-25]. Compared with miRNAs, piRNAs are less well conserved across taxa. Yet another class of small RNAs, endogenous small interfering RNAs (endo-siRNAs), are usually 21 nucleotides and have been found in some taxa, including nematodes [26], flies [27-29], and mammals [30,31], but it has not been well characterized in other animals.

Evolutionary genetic studies examining small RNAs are important for several reasons. First, genome-wide allele-specific mRNA expression analyses have revealed that both *cis*- and *trans*-regulatory changes contribute to differential expression of mRNAs among closely related species [32-34]. Small RNAs can act as *trans*-regulatory factors, which contribute to differential mRNA expression [35]. Additionally, *cis*-regulatory changes may include mutations at the target sites of small RNAs [36]; for example, SNPs and insertion-deletion polymorphisms were identified within miRNA-binding sites of 3’-untranslated regions [37,38]. Variations in small RNA transcriptomes and sequences were found to be associated with phenotypic variation in humans and laboratory animals. For example, miRNA and miRNA target site polymorphisms and mutations have been found in humans and are associated with disease susceptibility [39-42]. Polymorphism in a miRNA target site is associated with variation of muscularity in pigs [43]. Second, small RNAs regulate translation of mRNAs. Therefore, transcriptome studies of mRNA alone can overlook the divergence in the total outcome of gene expression among species. Third, piRNAs may contribute to hybrid abnormalities (see above), but generalities regarding the roles of piRNA in different types of hybrid abnormalities remain unclear.

In the present study, we compared brain small RNA transcriptomes between incipient species of the threespine stickleback (*Gasterosteus aculeatus*). The threespine stickleback is a good model for linking ecological and genetic studies of adaptive evolution and speciation [44-52]. The threespine stickleback has undergone tremendous diversification over the past few million years [44,45,49]. Evolutionary diversification within the stickleback species complex led to a speciation continuum, which ranges from populations with interspecific phenotypic polymorphism to strong divergence with near-complete reproductive isolation [44,53]. Recent genetic studies have revealed that differences in the expression of genes involved in morphological development [54,55], physiology [56,57], and immune function [58] may underlie adaptive divergence among populations or species. Sex bias of the mRNA transcriptome has also been investigated, and genes located on sex chromosomes were found to be female-biased, likely owing to Y-chromosome degeneration and lack of dosage compensation [59]. However, transcriptome analysis of small RNAs has not yet been conducted in any stickleback system.

This study focused on Japanese threespine stickleback species pairs, including a Pacific Ocean form and a Japan Sea form. These sticklebacks diverged during a period of geographical isolation between the Sea of Japan and the Pacific Ocean approximately 1.5–2 million years ago [60,61]. Currently, they are sympatric in eastern Hokkaido, but they are reproductively isolated with a very low level of hybridization [60-62]. In the Japan Sea form, a chromosomal fusion occurred between linkage group (LG) 9 and the ancestral Y chromosome (LG 19), resulting in the evolution of the X_1_X_2_Y multiple sex chromosome system [62]. In contrast, the Pacific Ocean form has a simple XY sex chromosome system [62]. Previously, we found that the Pacific Ocean and Japan Sea forms diverge in male courtship behaviors and female mate choice behaviors, contributing to behavioral isolation between these two forms [60,62,63]. Furthermore, we found that divergence in the intensity of courtship behaviour, which is important for mate choice, mapped to a neo-sex chromosome (LG 9).

To better understand the genetic mechanisms affecting behavioral differences between this Japanese stickleback species pair, it is essential to understand divergence in small RNA transcriptomes of the brain. Both miRNAs and piRNAs play important roles in a diverse array of neuronal functions such as neuronal differentiation, neural stem cell renewal, neuronal outgrowth, and dendritic spine morphogenesis [23,24,64]. Furthermore, variation in miRNA expression patterns in the brain may contribute to behavioral differences among laboratory mouse strains [65]. Additionally, in the Japanese stickleback system, courtship dysfunction may exist in hybrids because a substantial number of hybrids did not perform any courtship behavior in a previous experiment (Supplementary data in [61]). Therefore, it is necessary to examine whether small RNAs, especially piRNAs, which may be regulating transposon activity in the brain, affect hybrid behavior. Here, we characterized the divergence in small RNA transcriptomes in the brain between the species pairs of Japanese threespine stickleback.

## Results and discussion

### miRNA transcriptome

**Figure 1:**
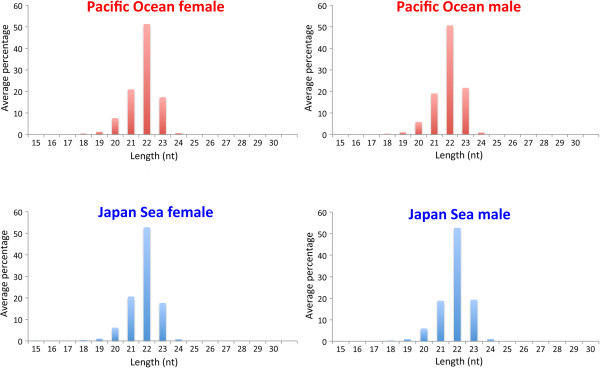
**Size distribution of stickleback brain miRNAs.** The average of four individuals is shown for each group.

**Figure 2:**
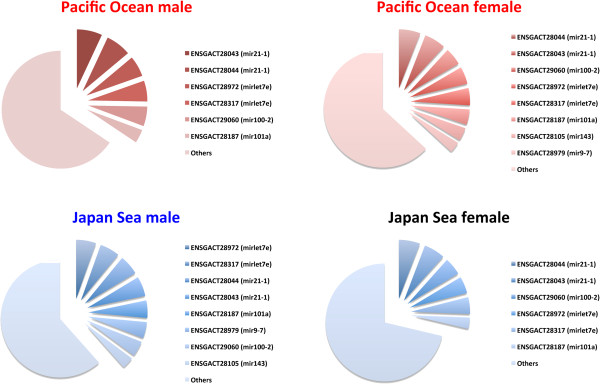
**miRNA expression profile in threespine stickleback brains.** The area indicates the fraction of read numbers of particular miRNAs among the total read number of annotated miRNAs. Only miRNAs whose expression is higher than 3% of all annotated reads are shown. The average of four individuals is shown for each group. Homologous zebrafish miRNA (blast, E < 10^-3^) are shown in parentheses.

We conducted small RNA sequencing of four males and four females from both the Pacific Ocean and the Japan Sea forms using the Illumina Genome Analyzer IIx. After quality control of the sequence reads, data was collected from 26.2 ± 3.3 (mean ± SD) million reads per fish (Additional file 1: Table S1). Most of these reads (23.7 ± 2.9 million reads; 86.9–94.6% of the total reads) were located in the Ensembl stickleback miRNA database. More than 50% of these small RNAs were 22 nucleotides in length (Figure 1). In total, 1300 isoforms of miRNA were detected in the brain (924 in the Pacific Ocean males, 924 in the Pacific Ocean females, 916 in the Japan Sea males, and 884 in the Japan Sea females). miRNA expression profiles demonstrated that miRNAs homologous to *mir21*, *mir100*, *let7*, *mir101*, *mir143*, and *mir9* were the most abundant in the stickleback brain, regardless of the species or sex (Figure 2). Most other miRNAs were expressed at relatively low levels (less than 3% of the annotated reads).

**Figure 3:**
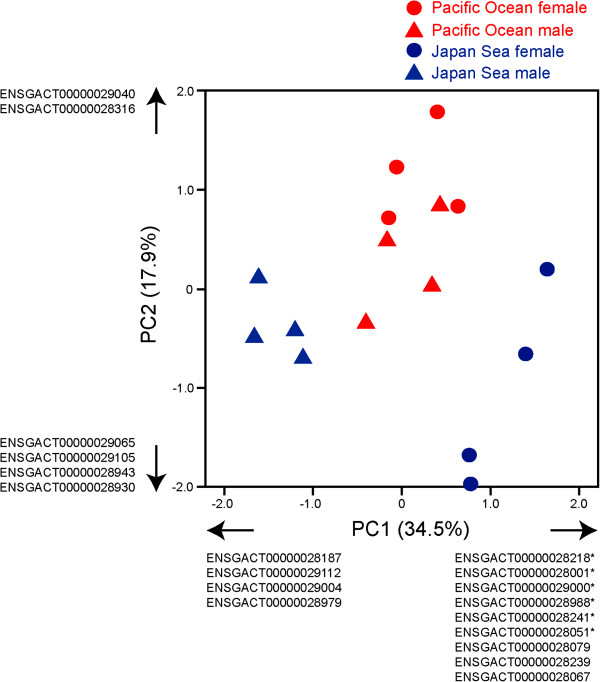
**Principal component analysis (PCA).** The first PC (PC1) and second PC (PC2) explained 34.5 and 17.9% of the variances, respectively. The loading components are shown in Table S2. The transcripts with loading component values larger than 0.75 and 0.70 for PC1 and PC2, respectively, are shown in the figure. The asterisks indicate the transcripts that were differentially expressed between the sexes by ANOVA with Bonferroni correction (Table 2).

To elucidate the variation in the miRNA transcriptomes, we conducted principal component analysis (Figure 3; Additional file 2: Table S2). The miRNA transcriptomes of the Pacific Ocean and the Japan Sea forms make distinct clusters. Interestingly, the Japan Sea males and females make distinct clusters, whereas the miRNA transcriptomes of the Pacific Ocean males and females overlapped. These data suggest that the magnitude of sex differences of miRNA expression levels might differ between species.

We identified several miRNAs that were differentially expressed between species (Bonferroni correction of analysis of variance [ANOVA]; Tables 1 and 2). Although quantitative trait loci (QTL) mapping revealed that LG9 contained a courtship display QTL, no miRNAs expressed from LG9 showed significantly different expression levels between species after Bonferroni correction (Table 1). We identified a miRNA homologous to the zebrafish *mir7* that was differentially expressed between the stickleback species. In mammals, *mir7* expression levels in the brain can change after hyperosmolar stimuli [66] and regulate growth factor signalling pathways [67]. Another miRNA differentially expressed between species, *mir30*, may be involved in axon guidance [68].

**Table 1:** miRNA differentially expressed between species

**Transcript ID**	**LG**	**Start position**	**Median reads per million (RPM)** ± **S**.**D**.				**Zebrafish homolog**
			**Pacific Ocean males**	**Pacific Ocean females**	**Japan Sea males**	**Japan Sea females**	
ENSGACT00000029029	I	7684495	2852.3 (227.8)	4343.0 (1716.7)	10218.3 (1270.0)	4026.2 (860.8)	*mir22a*-*1*
ENSGACT00000028961	III	7312510	563.3 (78.4)	1250.9 (684.9)	3683.1 (408.8)	1115.0 (176.9)	*mir7a*-*3*
ENSGACT00000028970	VI	4231011	2465.3 (127.5)	2270.1 (62.9)	1858.6 (244.0)	1966.8 (141.3)	*mir30c*
ENSGACT00000029035	XI	1952924	9700.4 (165.6)	11004.3 (1503.8)	14871.2 (475.2)	12259.0 (332.2)	*mir152*
ENSGACT00000028984	XIII	7256736	609.2 (80.5)	1294.3 (679.4)	3601.1 (393.0)	1134.8 (168.2)	*mir7b*
ENSGACT00000029072	XIX	2936579	547.6 (75.4)	1228.1 (681.4)	3503.5 (381.2)	1073.8 (169.6)	*mir7a*-*1*
ENSGACT00000029039	XX	15698389	568.3 (73.6)	1245.5 (680.7)	3560.3 (396.7)	1099.6 (167.8)	*mir7a*-*2*

*LG*: linkage group.

**Table 2:** ANOVA of miRNA

**Transcript ID**	**LG**	**Species**		**Sex**		**Species X Sex**	
		***F***	***P***	***F***	***P***	***F***	***P***
ENSGACT00000029029	I	**34**.**2**	<**0**.**001**	5.9	0.032	**46**.**1**	<**0**.**001**
ENSGACT00000028961	III	**44**.**5**	<**0**.**001**	8.8	0.012	**39**.**6**	<**0**.**001**
ENSGACT00000028970	VI	**25**.**9**	<**0**.**001**	0.1	0.782	2.9	0.115
ENSGACT00000029035	XI	**57**.**6**	<**0**.**001**	0.9	0.353	**23**.**8**	<**0**.**001**
ENSGACT00000028984	XIII	**42**.**6**	<**0**.**001**	8.7	0.012	**39**.**8**	<**0**.**001**
ENSGACT00000029072	XIX	**41**.**2**	<**0**.**001**	8.0	0.015	**38**.**2**	<**0**.**001**
ENSGACT00000029039	XX	**42**.**1**	<**0**.**001**	8.4	0.013	**38**.**2**	<**0**.**001**
ENSGACT00000029075	IV	4.6	0.053	**50**.**3**	<**0**.**001**	0.0	0.926
ENSGACT00000028051	IV	0	0.971	**84**	<**0**.**001**	**29**.**2**	<**0**.**001**
ENSGACT00000028218	VII	0	0.852	**77**.**9**	<**0**.**001**	**26**.**1**	<**0**.**001**
ENSGACT00000028988	XII	0	0.97	**83**.**1**	<**0**.**001**	**28**.**8**	<**0**.**001**
ENSGACT00000028001	XVII	0	0.956	**76**.**5**	<**0**.**001**	**27**.**9**	<**0**.**001**
ENSGACT00000029000	XVII	0	0.966	**83**.**2**	<**0**.**001**	**28**.**9**	<**0**.**001**
ENSGACT00000029064	XIX	5.1	0.043	**51**	<**0**.**001**	0.0	0.963
ENSGACT00000028241	XIX	0	0.976	**84**.**3**	<**0**.**001**	**29**.**1**	<**0**.**001**

*LG*: linkage group.

Bold letters indicate significance even after Bonferroni correction (*P* < 0.0003).

**Figure 4:**
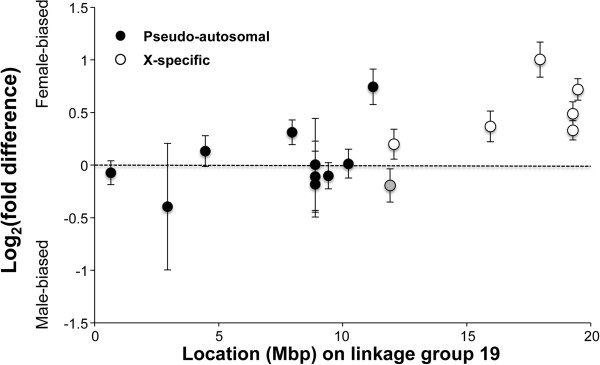
**Female**-**biased expression of miRNA expressed from the non**-**recombining region of the X chromosome (linkage group 19).** Black circles indicate small RNAs on the pseudoautosomal region, whereas white circles indicate small RNAs from the X-specific region. A small RNA, for which it was not clear whether the RNA was located on the pseudoautosomal or X-specific region, is indicated by a grey circle. For statistical analysis, the grey circle was excluded. Data from the Japan Sea and Pacific Ocean fish were pooled. Because the order of the LG19 sequence assembly on the ensembl is inverted after 3.822 Mbp, physical locations on LG19 followed [62]. Error bars indicate S.E.

Sex differences in the expression levels were identified for several miRNAs (Tables 2 and 3). Interestingly, all sex-biased miRNAs belonged to the *let*-*7* family (Table 3) [69]. miRNAs of the *let*-*7* family are highly conserved across taxa and are important during development [70,71]. Two sexually dimorphic miRNAs (ENSGACT00000028241 and ENSGACT00000029064) were expressed from LG19; both of these were female-biased. In species with an XY-sex chromosome system, suppression of recombination can lead to degeneration of the Y chromosome [72,73]. Unless dosage compensation mechanisms evolve, expression of genes located on the X-specific region becomes female-biased [74]. Therefore, we investigated the relationship between Y-degeneration and sex differences in miRNA expression levels. We found that expression of all miRNAs derived from the X-specific region (i.e. the counterpart region of the Y is likely degenerated) were female-biased, whereas expression of miRNA derived from the pseudoautosomal region was not necessarily female-biased (Figure 4), and log_2_ (fold difference between male and female) significantly differed between the pseudo-autosomal and the X-specific regions (Mann–Whitney *U* test, *U* = 7, *Z* = 814.5, *P* < 0.001, *N* = 17). These results suggest that Y chromosome degeneration may have a substantial impact not only on mRNA expression [59], but also on miRNA expression.

**Table 3:** miRNA differentially expressed between males and females

**Transcript ID**	**LG**	**Start position**	**Median reads per million (RPM) ± S.D.**				**Zebrafish homolog**
			**Pacific Ocean males**	**Pacific Ocean females**	**Japan Sea males**	**Japan Sea females**	
ENSGACT00000029075	IV	20044816	1606.0 (181.4)	2616.6 (434.6)	1421.9 (60.4)	2339.2 (396.8)	*mirlet7i*
ENSGACT00000028051	IV	19861309	20839.3 (1126.5)	22453.2 (1081.8)	18010.6 (611.4)	25558.9 (1427.5)	*mirlet7a*-*3*
ENSGACT00000028218	VII	18314432	19933.6 (1139.6)	21367.4 (1035.2)	17101.4 (549.5)	24133.6 (1426.5)	*mirlet7a*-*4*
ENSGACT00000028988	XII	12165589	20768.4 (1137.2)	22362.8 (1085.0)	17924.8 (600.1)	25445.9 (1434.9)	*mirlet7a*-*6*
ENSGACT00000028001	XVII	11962085	20520.0 (1120.3)	21925.0 (1071.1)	17736.6 (610.0)	24909.7 (1419.4)	*mirlet7a*-*1*
ENSGACT00000029000	XVII	3393574	20777.3 (1135.5)	22362.4 (1084.3)	17935.0 (602.4)	25456.6 (1431.4)	*mirlet7a*-*6*
ENSGACT00000029064	XIX	4582643	1608.3 (184.2)	2633.7 (436.6)	1411.8 (66.8)	2327.6 (395.7)	*mirlet7i*
ENSGACT00000028241	XIX	4785031	20810.0 (1125.6)	22433.4 (1083.4)	17991.0 (609.0)	25531.1 (1426.0)	*mirlet7a*-*3*

*LG*: linkage group.

Because miRNAs can target many mRNAs [75,76], divergence in miRNA expression patterns may have substantial effects on the expression patterns of many mRNAs. Further experimental studies examining the roles of small RNAs in fish will be necessary to understand the functional effects of the miRNA transcriptome variation. This would be possible by developing either transgenic fish specifically overexpressing small RNAs or small RNA-deficient knockout fish [77-81].

### Small RNAs homologous to repetitive sequences

**Figure 5:**
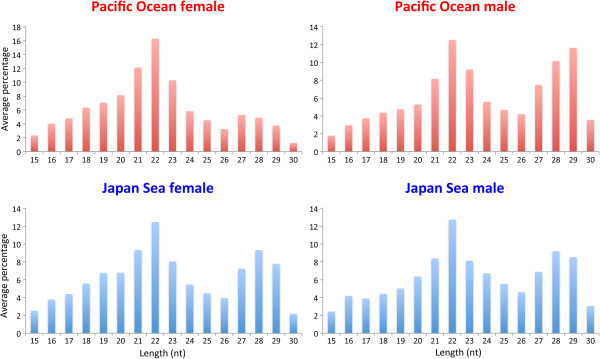
**Size distribution of non**-**annotated small RNAs.** The average of four individuals is shown for each group.

**Figure 6:**
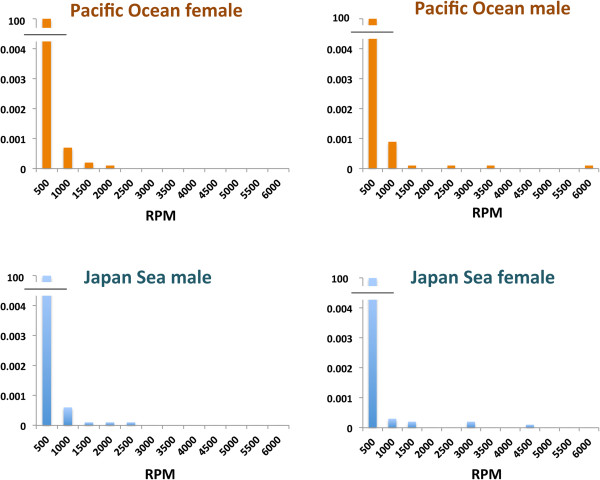
**Histogram of reads per million (RPM) of non**-**annotated small RNAs.** The average of four individuals is shown for each group.

Small RNAs with no matches in the stickleback non-coding RNA database were further analyzed. A histogram of the read length of these small RNAs revealed two peaks, with one peak at 22 nucleotides and the other at 27–29 nucleotides (Figure 5). The fraction of large-sized small RNAs may contain piRNAs, whereas the other fraction may correspond to novel miRNAs and/or endo-siRNAs. A histogram of reads per million (RPM) of the unidentified small RNAs revealed that most were expressed at low levels, with only a few expressed at high levels (Figure 6). Thirty-one novel small RNAs expressed at relatively high abundance (mean RPM > 50 in at least one of the four groups) could be classified into 17 isoforms on the basis of sequence identity (Additional file 3: Table S3).

A homology search against the piRNABank database revealed that some of these were similar to previously reported piRNAs (Table 4). Additionally, seven isoforms contained 5^′^U (T in Table 4), which is often found in previously reported piRNAs. However, compared with miRNAs, piRNAs are less conserved across taxa. Therefore, we examined whether these 17 small RNAs showed homology to repetitive sequences such as transposons. For all 17 isoforms, multiple homologous sites were identified in the stickleback genome (Table 5). Most of these potential small RNA target sites overlapped with repetitive sequences (Tables 5 and Additional file 4: Table S4). Four isoforms (iso-smRNA6, 9, 12, and 13) showed a high level of homology to the non-long terminal repeat (non-LTR) retrotransposon. One isoform (iso-smRNA5) was homologous to the LTR retrotransposon and two isoforms (iso-smRNA11 and 17) were homologous to ERV1-type retrovirus genes. One (iso-smRNA8) was similar to a DNA transposon. Because we did not confirm that these sequences actually bind to Piwi proteins, we could not exclude the possibility that the identified sequences are not piRNAs. However, all of these were longer than 24 nucleotides, and some of them contained the 5^′^U. Recent studies have demonstrated that retrotransposons are active in the adult mammalian brain and are thought to increase neuronal function diversity [82]. Therefore, regardless of whether these sequences are piRNAs, it is interesting that small RNAs highly homologous to transposons are expressed in the stickleback brain.

**Table 4:** Small RNAs with nucleotide lengths larger than 25 nt

**smallRNA ID**	**Sequence**	**Length (nt)**	**Hit in the piRNABank**	**E-value**
iso_smRNA1	CCCTCGGTTCTGGCGTCAAGCGGGCCGGC	29	No hit	-
iso_smRNA2	GCATGTGGTTCAGTGGTAGAATTCTCG	27	hsa_piR_018570	0.0053
iso_smRNA3	GCATTGGTGGTTCAGTGGTAGAATTCTCGC	30	dr_piR_0029993	0.0000065
iso_smRNA4	GCATTGTGGTTCAGTGGTAGAATTCTCGCC	30	hsa_piR_018570	0.00065
iso_smRNA5	GCCCGGCTAGCTCAGTCGGTAGAGCATGA	29	hsa_piR_000794	0.000017
iso_smRNA6	GGGTTCGATTCCCGGTCAGGGAACCA	26	No hit	-
iso_smRNA7	GGTTCCATGGTGTAATGGTTAGCACTCTG	29	hsa_piR_020582	0.000019
iso_smRNA8	GGTTCTATGGTGTAATGGTTAGCACTCTG	29	hsa_piR_020582	0.00011
iso_smRNA9	GTTGTCGTGGCCGAGTGGTTAAGGCAATG	29	hsa_piR_015249	0.0054
iso_smRNA10	GTTTCCGTAGTGTAGTGGTTATCACGTTCG	30	rno_piR_005901	0.0000065
iso_smRNA11	TCCCATATGGTCTAGCGGTTAGGATTCCT	29	dr_piR_0027014	0.018
iso_smRNA12	TCCCTGGTGGTCTAGTGGTTAGGATTCGGC	30	ona_piR_166322	0.0000065
iso_smRNA13	TCCCTGTGGTCTAGTGGTTAGGATTCGGCG	30	ona_piR_166322	0.00049
iso_smRNA14	TCCTCGTATAGTGGACAGTATCTCCGCC	28	No hit	-
iso_smRNA15	TGAAAGACAACTCTTAGCGGTGGATC	26	No hit	-
iso_smRNA16	TGCGACCTCAGATCAGACGAGACAACCC	28	dr_piR_0026826	0.0056
iso_smRNA17	TGGCTTCCTAAGCCAGGGATTGTGGG	26	No hit	-

E-value smaller than 0.05 is shown here.

**Table 5:** Characterization of small RNAs with high homology to repetitive sequences

**smallRNA ID**	**Length (nt)**	**Blast hits** (**E** <**10**^-**4**^)	**Potential target repeat**	**Description**	**Median reads per million** (**RPM**) ± **S**.**D of the longest isoform**			
					**Pacific Ocean male**	**Pacific Ocean female**	**Japan Sea male**	**Japan Sea female**
iso_smRNA1	29	scaffolds(7)	LSU-rRNA_Mfr	rRNA	72.6 (17.5)	56.9 (43.6)	58.9 (11.4)	22.6 (9.8)
iso_smRNA2	27	LG1(12)	tRNAGlyGGC_CB	tRNA	64.2 (20.9)	51.9 (71.8)	56.9 (101.1)	25.5 (13.7)
iso_smRNA3	30	LG1(12), LG12(1)	tRNAGlyGGC_CB	tRNA	52.6 (7.5)	34.4 (143.5)	91.1 (32.1)	18.1 (8.7)
iso_smRNA4	30	LG1(12), LG12(1)	tRNAGlyGGC_CB	tRNA	308.0 (161.0)	131.8 (611.1)	729.2 (366.5)	179.7 (105.6)
iso_smRNA5	29	LG1(2), LG7(2), LG8(3), LG10(1), LG11(7), LG15(1), LG16(1), LG17(1), scaffolds(86)	Gypsy-14_DAn-I	LTR retrotransposon	304.6 (162.0)	120.7 (572.5)	51.5 (266.1)	22.0 (30.8)
iso_smRNA6	26	LG3(1), LG7(1), LG11(2), LG12(1), LG17(5), LG18(1), LG19(1), LG20(1)	SINE2-1_EC	Non-LTR Retrotransposon	79.0 (29.0)	67.7 (28.7)	83.7 (30.3)	62.3 (23.4)
iso_smRNA7	29	LG3(54), LG5(1), LG9(1), LG11(2), LG12(1), LG13(1), LG17(1), LG19(1), LG20(32), scaffolds(30)	tRNA-Val-GTA	tRNA	86.9 (22.0)	57.2 (123.0)	25.7 (16.6)	12.7 (10.8)
iso_smRNA8	29	LG5(1), LG9(1), LG11(2), LG12(1), LG13(1), LG17(1)	DNA-TTAA-5_NV	DNA transposon	219.9 (50.9)	138.8 (312.8)	53.7 (47.9)	41.5 (23.3)
iso_smRNA9	29	LG8(1), LG9(1), LG11(1), LG12(1), LG13(1), LG16(1), scaffolds(5)	SINE2-8_SP	Non-LTR Retrotransposon	72.0 (10.9)	58.4 (61.2)	15.2 (17.9)	9.9 (12.2)
iso_smRNA10	30	LG7(6), LG18(10), scaffolds(44)	tRNA-Val-GTA	tRNA	309.1 (78.0)	150.1 (701.4)	581.7 (417.1)	153.0 (99.2)
iso_smRNA11	29	LG1(1), LG4(1), LG7(1), LG12(1), scaffold(1)	LTR10A2_SS	ERV1-type endogenous retrovirus	292.2 (76.3)	177.0 (434.9)	364.8 (124.6)	155.6 (74.5)
iso_smRNA12	30	LG3(1), LG7(1), LG11(2), LG12(1), LG17(5), LG18(1), LG19(1), LG20(1)	SINE2-1_EC	Non-LTR Retrotransposon	94.4 (21.8)	29.9 (90.1)	51.3 (19.5)	19.9 (7.5)
iso_smRNA13	30	LG3(1), LG7(1), LG11(2), LG12(1), LG17(5), LG18(1), LG19(1), LG20(1)	SINE2-1_EC	Non-LTR Retrotransposon	135.5 (47.2)	43.5 (124.1)	101.0 (54.8)	35.9 (12.3)
iso_smRNA14	28	LG7(7), LG12(1)	tRNA-Asp-GAY	tRNA	86.0 (16.3)	68.4 (51.3)	140.7 (166.9)	74.8 (25.0)
iso_smRNA15	26	scaffolds(10)	LSU-rRNA_Mfr	rRNA	142.1 (40.6)	122.1 (42.0)	193.3 (64.9)	69.9 (27.3)
iso_smRNA16	28	scaffolds(10)	LSU-rRNA_Mfr	rRNA	140.7 (52.4)	170.6 (82.9)	140.1 (29.1)	74.3 (26.3)
iso_smRNA17	26	LG12(1)	LTR41_SS	ERV1-type endogenous retrovirus	76.8 (3.3)	57.2 (20.0)	64.1 (35.9)	41.0 (5.8)

The numbers in parentheses in the Blast hits indicate the number of hits on that linkage group.

The remaining six and three isoforms overlapped with repetitive sequences homologous to tRNA and rRNA, respectively. Previous studies also identified a number of tRNA-derived small RNAs in humans [83,84], *Giardia lamblia*[85], and zebrafish [86]. These tRNA-derived small RNAs may contribute to gene regulation [84], although little is yet known about their functions. Interestingly, some transposons are derived from tRNAs [87,88], so there appears to be an interesting link between tRNA and small RNAs.

Finally, we found that the iso-smRNA9, whose potential targets are predicted to be non-LTR transposons, was more highly expressed in the Japan Sea sticklebacks than in the Pacific Ocean sticklebacks (ANOVA, *F*_1, 13_ = 14.5, *P* = 0.002). Although expression levels of some other piRNAs may differ between different species and sexes, the differences were not significant after Bonferroni correction (Additional file 5: Table S5). None of the non-annotated small RNAs showed sex differences in the expression levels (Additional file 5: Table S5).

Thus, we identified small RNAs homologous to repetitive sequences such as RNA and DNA transposons. Hybrids between species often exhibit courtship dysfunction [89-94]. Abnormal transposon activity in hybrids may cause hybrid courtship dysfunction, but this has not been tested in any organism. Intra- and inter-population variation in the presence and absence of non-LTR retrotransposons has been found in sticklebacks [95]. In addition, our analysis involving whole genome sequence comparisons also revealed that DNA transposon insertion sites diverge between the Pacific Ocean and the Japan Sea sticklebacks (Kitano, unpublished data). Therefore, further studies examining variation in transposon activity between the different species and transposon activity in the hybrids will lead to a better understanding of speciation mechanisms.

## Conclusions

Our study demonstrates that closely related species can show divergence in expression patterns of small RNAs, including miRNAs and piRNAs. Some of the sex differences in miRNA expression levels might result from Y-chromosome degeneration. Therefore, variation in small RNA transcriptomes should be examined as a potential mechanism underlying phenotypic divergence between incipient species.

## Methods

### Small RNA sequencing

Sympatric Pacific Ocean and Japan Sea sticklebacks were collected using minnow traps from the Bekanbeushi River System on Hokkaido Island, Japan in June 2007 [60,62]. Fish were brought to a laboratory to examine the brain transcriptome of courting male and spawning female fish, and mating experiments were conducted in June and July 2007, as described previously [60,63,96]. Once the male fish had constructed a nest, a conspecific gravid female fish was placed in the same tank. Immediately after the female fish had inspected the nest, both the male and the female fish were removed from the tank prior to spawning. After immersing the fish in a lethal dose of tricaine methanesulfonate, the brains of each fish were dissected and stored separately at −70°C.

For RNA sequencing, we used four Pacific Ocean male, four Pacific Ocean female, four Japan Sea male, and four Japan Sea female fish (*N* = 16 in total). Total RNA was isolated using TRIzol Reagent (Life Technologies, Grand Island, NY, USA), and the quality of RNA was evaluated using the BioAnalyzer (Agilent, Santa Clara, CA, USA). RNA Integrity Number (RIN) ranged from 9.3 to 10 with the median of 10. Libraries were constructed using the TruSeq Small RNA Sample Preparation Kit (Illumina, San Diego, CA, USA). Small RNAs with 20–30 nucleotides were isolated according to the manufacturer’s instructions. Sequencing was performed using the Genome Analyzer IIx (Illumina, San Diego, CA, USA) at the University of Tokyo. We used 16 lanes of the Genome Analyzer IIx (one fish per one lane).

### miRNA analysis

Sequence analyses were conducted using the CLC Genomics Workbench Software (CLC bio, Katrinebjerg, Denmark). First, we discarded reads with a low quality score (quality score on the Phred scale of less than 0.05), very short length (less than 14 bp), or three or more ambiguous nucleotides (Additional file 1: Table S1). Next, identical reads were clustered together to group different types of small RNAs. Next, the sequences were mapped against the Ensembl stickleback miRNA database (http://asia.ensembl.org/info/data/ftp/index.html) with two nucleotides mismatches allowed.

Principal component analysis (PCA) of reads per million (RPM) was conducted on a Pearson correlation matrix. To identify differentially expressed miRNAs between various species and sexes, statistical analyses were conducted on the square-root transformed RPM of each miRNA. Using the statistical package R [97], analysis of variance (ANOVA) was conducted to examine whether the species, sex, and their interactions significantly influenced RPM. Because the patterns of sex differences varied between species (see Figure 3), we included the interaction term for the analysis. The Bonferroni correction was used for multiple comparison correction. Shapiro-Wilk and Bartlett’s tests were used to test for normal distribution of the data and homogeneity of variances, respectively. None of the miRNAs violated the assumptions of the normal distribution or homogeneity of variances after Bonferroni correction. Only miRNAs with a mean RPM higher than 1000 in at least one of the four groups (Pacific Ocean male, Pacific Ocean female, Japan Sea male, or Japan Sea female) were used for PCA and ANOVA.

### piRNA analysis

Small RNAs with no match to any sequences in the stickleback miRNA database were mapped against sequences in the Ensembl stickleback non-coding RNA database (http://asia.ensembl.org/info/data/ftp/index.html), which includes transfer RNA (tRNA), ribosomal RNA (rRNA), small cytoplasmic RNA, small nuclear RNA, small nucleolar RNA, microRNA precursors, long intergenic non-coding RNAs, and other miscellaneous RNA. The parameters used were the same as above. Small RNAs with no match to any sequences in the stickleback non-coding RNA database may contain piRNAs; thus, we further analyzed these small RNAs to identify stickleback piRNAs. We first examined non-annotated small RNAs with nucleotide lengths >25 because this fraction is more likely to contain piRNAs. Among these longer small RNAs, we examined small RNAs with a mean RPM higher than 50 in at least one of the four groups (Pacific Ocean male, Pacific Ocean female, Japan Sea male, or Japan Sea female). Although some small RNAs showed differences in length, identical sequences were observed (Additional file 3: Table S3); hence, identical reads of different sizes were considered to be the same isoform.

To examine whether these small RNAs contain homologous sequences against any repetitive sequences, the longest isoforms were blasted against the stickleback genome (BROADS 1.56) using the default parameters (match = 1; mismatch = −3; gap existence = 5; gap extension = 2) of the CLC Genomics Workbench software. Next, flanking sequences (3,000 bp of upstream and 3,000 bp of downstream) were downloaded using Perl script [98]. We then examined whether these regions contained any repetitive sequences using the CENSOR software [99] on the Genetic Information Research Institute website (http://www.girinst.org/). When hits of repetitive sequences were identified in the region, we investigated whether the small RNA sequences overlapped with repetitive sequences. We also blasted these sequences against the piRNABank database [100].

To identify differentially expressed piRNAs between different species and sexes, statistical analysis was conducted on the square-root transformed RPM. Square-root transformed RPM values were subjected to ANOVA, followed by Bonferroni correction. Shapiro-Wilk and Bartlett’s tests were used to test for normal distribution of the data and homogeneity of variances, respectively. Two small RNAs, iso_smRNA3 and iso_smRNA14, did not meet the assumptions of homogeneity of variance (Bartlett’s test, *K*-squared = 15.2, *P* = 0.0017) and normal distribution (Shapiro-Wilk test, *W* = 0.779, *P* = 0.0014), respectively. For these small RNAs, we also conducted a Mann–Whitney *U*-test to confirm that these two small RNAs did not exhibit any differences between sexes and species. Because the interaction between the sexes and different species showed no significant effect for any of these small RNAs, the interaction term was excluded.

## Additional files

Additional files: **Tables S1-S5**. All of the short read sequences are deposited in the Sequence Read Archive (DRA) of the DNA Data Bank of Japan (DDBJ): accession number DRA000919.

## Abbreviations

miRNA: microRNA; mRNA: messenger RNA; piRNA: Piwi-interacting RNA; LG: linkage group; RPM: reads per million; PCA: principal component analysis; ANOVA: analysis of variance; tRNA: transfer RNA; rRNA: ribosomal RNA.

## Competing interests

The authors declare they have no competing interests.

## Authors’ contributions

JK carried out most of the experiments and analyses and wrote the manuscript. KY helped with the analyses. YS conducted RNA sequencing. All authors read and approved the final manuscript.

## Supplementary Material

Additional file 1: Table S1 Sequence raeds from each fish. Click here for file

Additional file 2: Table S2 Component loading of principal component analysis. Click here for file

Additional file 3: Table S3 Non-annotated small. Click here for file

Additional file 4: Table S4 Repetitive sequences homologous to small RNAs. Click here for file

Additional file 5: Table S3 ANOVA of non-annotated small RNAs. Click here for file
